# A Variant of the Histone-Binding Protein sNASP Contributes to Mouse Lupus

**DOI:** 10.3389/fimmu.2019.00637

**Published:** 2019-04-02

**Authors:** Jiyu Ju, Jia Xu, Yaoqiang Zhu, Xiaoyan Fu, Laurence Morel, Zhiwei Xu

**Affiliations:** ^1^Department of Immunology, Weifang Medical University, Weifang, China; ^2^Department of Pathology, Beth Israel Deaconess Medical Center, Harvard Medical School, Boston, MA, United States; ^3^Immunology and Laboratory Medicine, Department of Pathology, College of Medicine, University of Florida, Gainesville, FL, United States; ^4^Department of Anatomy and Cell Biology, College of Medicine, University of Florida, Gainesville, FL, United States

**Keywords:** mouse, lupus, lupus nephritis, genetics, NASP, histone-binding protein

## Abstract

The *Sle2c1rec1c* (*rec1c*) sublocus is derived from the mouse lupus susceptibility 2 (*Sle2*) locus identified in the NZM2410 model. Our current study dissected the functional characters and the genetic basis of the *rec1c* locus relative to lupus when co-expressed with the Fas^*lpr*^ mutation, an established inducer of autoimmunity. The rec1c.lpr mice exhibited mild expansion of lymph nodes and had a normal T cell cellularity, but developed significantly kidney and lung inflammation, indicating that the *rec1c* amplifies *lpr*-induced autoimmune pathogenesis. A variant of somatic nuclear autoantigenic sperm protein (sNASP) was identified from the *rec1c* interval as a substitution of two consecutive amino acid residues in the histone-binding domain, resulting in an increased binding affinity to histone H4 and H3.1/H4 tetramer. To determine the role of the *sNASP rec1c* allele in mouse lupus, a novel strain was generated by introducing the *rec1c* mutations into the B6 genome. In this transgenic model, the *sNASP* allele synergized with the *lpr* mutation leading to moderate autoimmune phenotypes and aggravating inflammatory pathology alterations in kidney and lung that were similar to those observed in the rec1c.lpr mice. These results establish that the *sNASP* allele is a pathogenic genetic element in the *rec1c* sublocus, which not only promotes autoimmunity, but also exacerbates the inflammation reaction of end organs in mouse lupus pathogenesis. It also shows the complexity of the *Sle2c* locus, initially mapped as the major locus associated with B1a cell expansion. In addition to *Cdkn2c*, which regulates this expansion, we have now identified in the same locus a protective allele of *Csf3r*, a variant of Skint6 associated with T cell activation, and now a variant of *sNASP* that amplifies autoimmunity and tissue damage.

## Introduction

Mouse models of systemic lupus erythematosus (SLE) have greatly contributed to the understanding of SLE pathogenesis, including by the identification of genetic pathways whose alterations lead to increased disease susceptibility or resistance (1). Although great efforts have been invested in the genetic analysis of spontaneous lupus mouse models, only a few lupus susceptibility genes have been identified with a putative causative etiology (2, 3). Although polymorphisms in these genes so far do not seem to be directly involved in human lupus, they fit into pathways that have been associated with lupus or other rheumatic diseases (2). The murine lupus susceptibility locus *Sle2* was identified on chromosome 4 as one of the three major loci associated with nephritis in the NZM2410 model (4). *Sle2* expression on a non-autoimmune background in the B6.Sle2 congenic strain revealed that it regulates B cell hyperactivity (5) and B1a cell expansion (6), but is not sufficient for clinical disease. However, the co-expression of *Sle2* with the *lpr* mutation in the Fas gene in the B6.Sle2.lpr mice resulted in more severe lupus nephritis and marked lymphadenopathy compared with B6.lpr mice (7).

The dissection of *Sle2* revealed a complex genetic architecture, with three independent loci, *Sle2a, Sle2b*, and *Sle2c*, contributing to B1a cell expansion, with the NZB-derived *Sle2c* being the strongest contributor (8). We identified a hypomorph allele of *Cdkn2c* as responsible for the *Sle2c* B1a cell expansion (9, 10). *Sle2c* contains a suppressive *Sle2c2* sublocus (11) that we have mapped to a missense mutation in the *Csf3r* gene encoding for the GCSF receptor and regulates the development of CD8a^+^ dendritic cells (11–13). In addition, we mapped the pathological phenotype synergizing with *lpr* to the centromeric portion of *Sle2c*, the *Sle2c1* sublocus (7). Subsequently, we generated a series of shorter *Sle2c1* intervals and investigated their epistatic interaction with *lpr* (14). Two non-overlapping subloci with non-redundant phenotypes were identified: The centromeric portion of *Sle2c1, Sle2c1rec1a*, which contains *Cdkn2c*, exerts a strong contribution to lupus autoimmunity without clinical phenotypes. A more telomeric sublocus named *Sle2c1rec1d* (*rec1d*) was associated with more severity of renal inflammation and lymphadenopathy, and higher frequency of dermatitis in the B6.Sle2c1rec1d.lpr (rec1d.lpr) mice than that in the B6.Sle2c1rec1a.lpr (rec1a.lpr) mice (14). It is reasonable to assume that the *Sle2c1rec1c* (*rec1c*) sublocus can contribute to mouse lupus by synergizing with the overlapping region of the *rec1a and rec1d* subloci, because the rec1d.lpr mice developed more severe autoimmune disease than rec1a.lpr mice (14).

**Figure 1:**
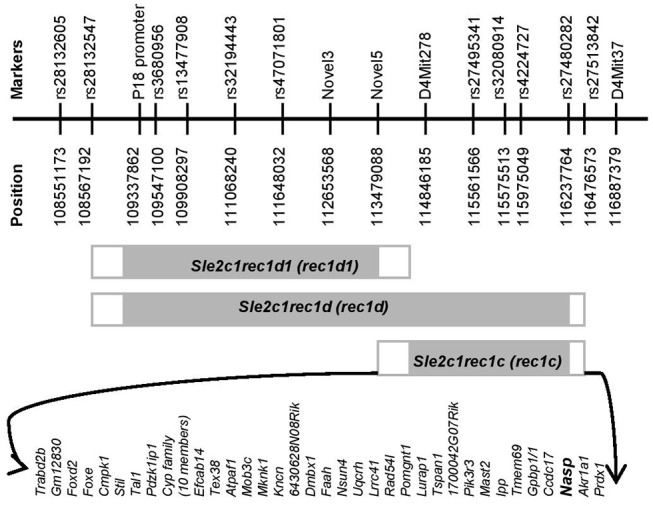
Physical map of the *rec1c* interval. The gray rectangles indicate the *Sle2c1* NZB-derived genomic fragments on the B6 genomic background located on mouse chromosome 4. The white rectangles highlight the areas of recombination between the B6 and NZB genomes. Fine mapping of the ends of each recombinant interval was performed by using markers that are polymorphic between the NZB and B6 genomes and are shown with names and positions at the top. The protein-encoding genes in the *rec1c* interval and its recombination area are displayed at the bottom. All mouse genome informatics used in this project were calculated from the NCBI m37 assembly.

Recently, we produced a novel recombinant *rec1d1* from the *rec1d* sublocus (Figure 1) that narrowed down the location of the gene responsible for the severe autoimmune disease with striking lymphadenopathy in the rec1d1.lpr mice (15). The only gene in the *rec1d1* interval that presented a non-synonymous mutation was *Skint6*, and this mutation resulted in a truncated secretory peptide (15). The *Skint6* protein is mainly expressed in mouse skin, and we obtained evidence that non-hematopoietic cells expressing the *rec1d1 Skint6* allele promoted T cell proliferation *in vivo*, suggesting that the *Skint6* variant is the most likely causal gene in the *rec1d1* sublocus.

The focus of this study was to analyze the phenotypes associated with the expression of *rec1c*, which is telomeric of *rec1d1* (14, 15). When combined with *lpr, rec1c* showed a modest effect on autoimmune phenotypes, and greatly aggravated kidney and lung pathology. Exon sequencing of all the coding genes present in the *rec1c* sublocus identified two non-synonymous mutations in the *rec1c* allele of the *sNASP* gene. The sNASP is the somatic isoform of the NASP protein, a histone-binding protein that controls H3.1 folding and regulates the pool of soluble H3-H4 histones available for DNA synthesis (16). NASP controls the progression through the cell cycle (17, 18) and regulates chromatin folding (19, 20). More recently, it has been shown that NASP regulates chromatin accessibility by maintaining a pool of H3K9me1 methylated histones (21), an epigenetic mark associated with active transcription sites (22). We show here that the *rec1c* allele of sNASP has a greater binding affinity for H4 histone or H3.1/H4 tetramers *in vitro*. Further, B6.lpr mice in which the mutated *sNASP* has been knocked-in displayed phenotypes similar to that of the rec1c.lpr mice, but also showed some extra autoimmune alterations. These results identify a gain of function allele of *sNASP* with the ability of increasing autoimmunity and aggravating inflammatory damage in end organs during lupus development. This discovery adds a new member to the list of pathogenic genes in murine lupus models and provides insights into new mechanisms of autoimmune diseases. It also identifies for the first time a natural variant of a histone-binding gene as a lupus susceptibility gene, potentially by regulating chromatin accessibility.

## Materials and Methods

### Mice

B6.lpr mice were purchased from Jackson Laboratory (Bar Harbor, ME, USA). The B6.Sle2c1rec1c.lpr strain was previously described (7). All available polymorphic genetic markers were used to refine the *rec1c* interval and define its ends. The B6.ΔsNASP mouse with the mutated bases of the *rec1c sNASP* allele introduced into the B6 genome was created by Cyagen Biosciences Inc. with the targeting strategy presented in **Figure 6A**. The mutated bases of the *rec1c sNASP* allele are in exon 12 of Nasp-001 ENSMUST00000030456. To construct the targeting vector, two homology arms were generated by PCR using BAC clone RP24-384F21 and RP24-72F14 from the C57BL/6J library as template. The CTGTACTCCATGAGC sequence in exon 12 of the *NASP* gene, corresponding to exon 10 of the *sNASP* isoform, was mutated to CT**A**TA**T**TCCATGAGC in the 5′ homology arm. In the targeting vector, a Neo cassette was flanked by Frt sites and DTA was used for negative selection. The constructed targeting vector was electroporated into C57BL/6 mouse embryonic stem (ES) cells, and then selected positive ES clones were microinjected into blastocysts. Chimeric mice were screened by genotyping and then bred with an Flp-deleter mouse to generate F1 mouse with constitutive knockin (KI) *rec1c sNASP* allele through Flp-mediated recombination. Finally, F1 mice were intercrossed to obtain a homozygous transgenic B6.ΔsNASP model. Following the previously described protocol (6), the *lpr* mutation was bred into the B6.ΔsNASP mouse to generate a B6.ΔsNASP.lpr strain. Both male and female mice were used in this study, without difference between genders. The protocols for mice used in this research were approved by the Institute Animal Care and Use Committees of the University of Florida, USA and Weifang Medical University, China.

### DNA Sequencing and RT-PCR

Genomic DNA of the lupus-prone strains MRL/MpJ-*Fas*^lpr^/J, NZM2410/J, BXSB/MpJ, NZB/B1NJ, and NZW/Lac/J was purchased from Jackson Laboratory. The Agilent SureSelect XT Mouse All Exon Capture Kit (Agilent Technologies, Inc., Santa Clara, CA, USA) used in this project has 50 Mb capture, covering the complete mouse exome and spanning over 221,784 exons and 24,306 genes. Mouse whole exome sequencing was performed by the Beijing Genomics Institute (Shenzhen, China), including DNA fragmentation, adapter ligation, hybridization with capture library, next-generation Illumina sequencing with an average 30x coverage, and bioinformatics analysis according to mouse genome assembly NCBIm37 (strain C57BL/6J). We selected the homozygous SNPs corresponding to non-synonymous mutations, frameshifts, deletions, insertions, stop loss or gain in coding regions. Total RNA was purified from tissues using the Qiagen RNeasy kit (Qiagen, Valencia, CA, USA) and converted into cDNA by reverse transcription using the SuperScript III First-Strand Synthesis System (Thermo Fisher Scientific, Waltham, MA). The Sanger method was used to sequence cDNA or specific exons. RT-PCR was utilized to semi-quantitatively detect sNASP mRNA expression (Forward primer: 5′ ACAAGCCCATCTTAAACTTGGAG3′; Reverse primer: 5′ CTGAGATTCCTTTGCGTCTTCTA 3′).

### Protein Expression, Purification, and Binding Kinetics of Protein Interaction

The full-length mouse *sNASP* cDNA (encoding 448 amino acids) was prepared from B6.lpr mouse using RT-PCR and then inserted into the pET30a expression vector to obtain a pET30a-WT sNASP protein expression vector. The mutated bases of the *rec1c sNASP* allele were introduced into the WT sNASP protein expression vector using the Q5® Site-Directed Mutagenesis Kit (NEB, Ipswich, MA) to generate a pET30a-*rec1c sNASP* allele protein expression vector. All *sNASP* constructs were confirmed by DNA sequencing. WT and mutated expression vectors were transformed into *E. coli* BL21(DE3)and protein expression was induced by Isopropyl β-D-1-thiogalactopyranoside (IPTG). Ion-exchange chromatography and size exclusion chromatography were used to purify proteins from the bacterial lysate. The protein purity was verified using SDS-PAGE electrophoresis. Western-blotting was used to identify mouse sNASP protein using anti-mouse NASP mAb (A-7, Santa Cruz Biotechnology, Inc., Dallas, TX).

Mouse histones H1a, H3.1, H4 were purchased from Lifespan Biosciences (Seattle, WA). The H3.1/H4 tetramer complex was prepared by incubating a mixture of mouse H3.1 and H4 overnight at room temperature followed by purification with size exclusion chromatography. The binding affinity of sNASP for histones was determined using biolayer interferometer Octet K2 system (Pall Fortebio Corp., Menlo Park, CA) at 30′C, following the instrument user guide. Briefly, aminopropylsilane (APS) biosensors were rinsed in assay buffer for 120 s to obtain an initial baseline. Next, mouse sNASP WT and mutant proteins were immobilized on the APS biosensors for 110 s to get a loading curve. Third, the sNASP-immobilized-APS biosensors were dipped into assay buffer for 120 s to acquire another baseline. Fourthly, the sNASP-immobilized-APS biosensors were exposed to various concentrations of histone H1a, H3.1, H4, and H3.1/H4 tetramer complex in assay buffer for 240 s to obtain association curves (K_on_/M^−1^s^−^1). Finally, the sNASP-immobilized-APS biosensors were again dipped into assay buffer without histones to get disassociation curves (K_off_/s^−1^). The interaction of mouse sNASP and mouse histones was expressed as layer thickness (nm) over time (second). The binding affinity (K_D_) was calculated by dividing K_on_ by K_off_. The protein expression, purification and measurements of binding kinetics were performed by Detai Biologics Company (Nanjing, China).

### IgG Autoantibody Detection

IgG anti-dsDNA and anti-chromatin IgG were measured by ELISA as previously described (6). Briefly, mBSA-coated plates were coated overnight with 50 mg/ml dsDNA for anti-dsDNA autoantibody detection. 10 mg/ml of histone H1, H2A, H2B, H3, and H4 were added to the dsDNA-coated plate for anti-chromatin autoantibody measurement. Test sera at 1:100 dilution was added to the plates and bound autoantibodies were detected using alkaline phosphatase-conjugated goat anti-mouse IgG and pNPP substrate. Raw optical densities were converted to units per milliliter, using a standard curve derived from pooled MRL/*lpr* serum, arbitrarily setting the reactivity of a 1:100 dilution of this serum to 100 U/ ml.

### Flow Cytometry

Cell subsets and activation status in spleen and lymph nodes were determined by flow cytometry as previously described (8). In brief, single-cell suspensions were prepared and depleted of red blood cells with 0.83% NH_4_Cl Tris-buffer. Cells were blocked with saturating amounts of anti-CD16/CD32 (2.4G2) and stained with fluorochrome-conjugated antibodies against CD3e (145-2C11), CD4 (RM4-5), CD69 (H1.2F3), CD44 (IM7). All antibodies were purchased from BD Pharmingen (San Jose, CA, USA) or eBioscience (San Diego, CA, USA). At least 50,000 events were acquired per sample using a FACSCalibur cytometer (BD Biosciences, San Jose, CA, USA).

### Kidney, Lung, and Liver Pathology

Tissues from 4 to 5-months-old mice were fixed and stained with hematoxylin and eosin (H&E). In addition, kidneys were also stained with periodic acid Schiff (PAS). Renal lesions were scored in a blinded manner following the previous report (7), and briefly speaking: grade 0, normal glomeruli, and evident capillary loops and unexpanded mesangium; grade 1, evident capillary loops, and widened mesangium with mild hypercellularity; grade 2, evident capillary loops, and expanded mesangium with more than moderate hypercellularity; grade 3, diminished capillary loops, swollen glomeruli, and more than 50% of all glomeruli with diffuse endocapillary proliferation; grade 4, no capillary loops, and basement membrane thickening and significant mesangial proliferation, more than 90% of all glomeruli with diffuse endocapillary proliferation. Lung pathology alterations were evaluated semi-quantitatively following the protocols in our publication (15), to briefly summarize: grade 0, normal lung architecture; grade 1, 1–10% of alveolar in lung has the pathological alterations of exudates, atelectasis and increased inflammatory cell number; grade 2, 10–25% of alveolar in lung shows the above pathological alterations and mild infiltrate of inflammatory cells around arteries and veins; grade 3, 25–50% alveolar in lung displays the above pathological alterations and moderate infiltrate of inflammatory cells around arteries and veins; grade 4, >50% alveolar in lung demonstrates the above pathological alterations and heavy infiltrate of inflammatory cells around arteries and veins.

The presence of immune complexes in the kidneys were evaluated on 5 μm frozen sections stained with FITC-conjugated rat anti-mouse C3 (SC-58926, Santa Cruz Biotechnology, Dallas, TX) and IgGκ BP-CFL 488 (SC-516176, Santa Cruz Biotechnology, Dallas, TX). Staining intensity was evaluated by examining sections with Olympus BX53 fluorescence microscope and DP80 camera (Diagnostic Instruments). Average 20 glomeruli for each sample was recorded as semi quantitative 0–4 scale using Image J software (NIH).

### Statistical Analysis

Data were analyzed with GraphPad Prism 5.0 software with the statistical tests indicated in the text. Non-parametric tests were used when data were not distributed normally.

## Results

### Fine-Mapping of the *rec1c* Sublocus

Since the *rec1*c interval is of NZB origin (8), we refined its map and defined its ends by genotyping all available markers that are polymorphic between the NZB and B6 genomes (Figure 1), including microsatellite Mit and single-nucleotide polymorphisms (SNPs) markers collected from the Mouse Genome Informatics (MGI), the National Center for Biotechnology Information (NCBI) or identified through our own genomic sequencing. The *rec1c* interval includes D4Mit278 at the centromeric end and rs27480282 at the telomeric end, but excludes the Novel5 marker and rs27513842, defining *rec1c* as a 1.39–2.99 Mb interval (Figure 1). The *rec1c* and *rec1d1* subloci do not overlap, but together cover the entire *rec1d* sublocus. The *rec1c* is in a gene-rich region, which contains 44 protein-coding genes, including those in the intersection area of B6 and NZB genomes (Figure 1).

### The *rec1c* Sublocus Promotes End Organ Inflammation in the rec1c.lpr Mouse

**Figure 2:**
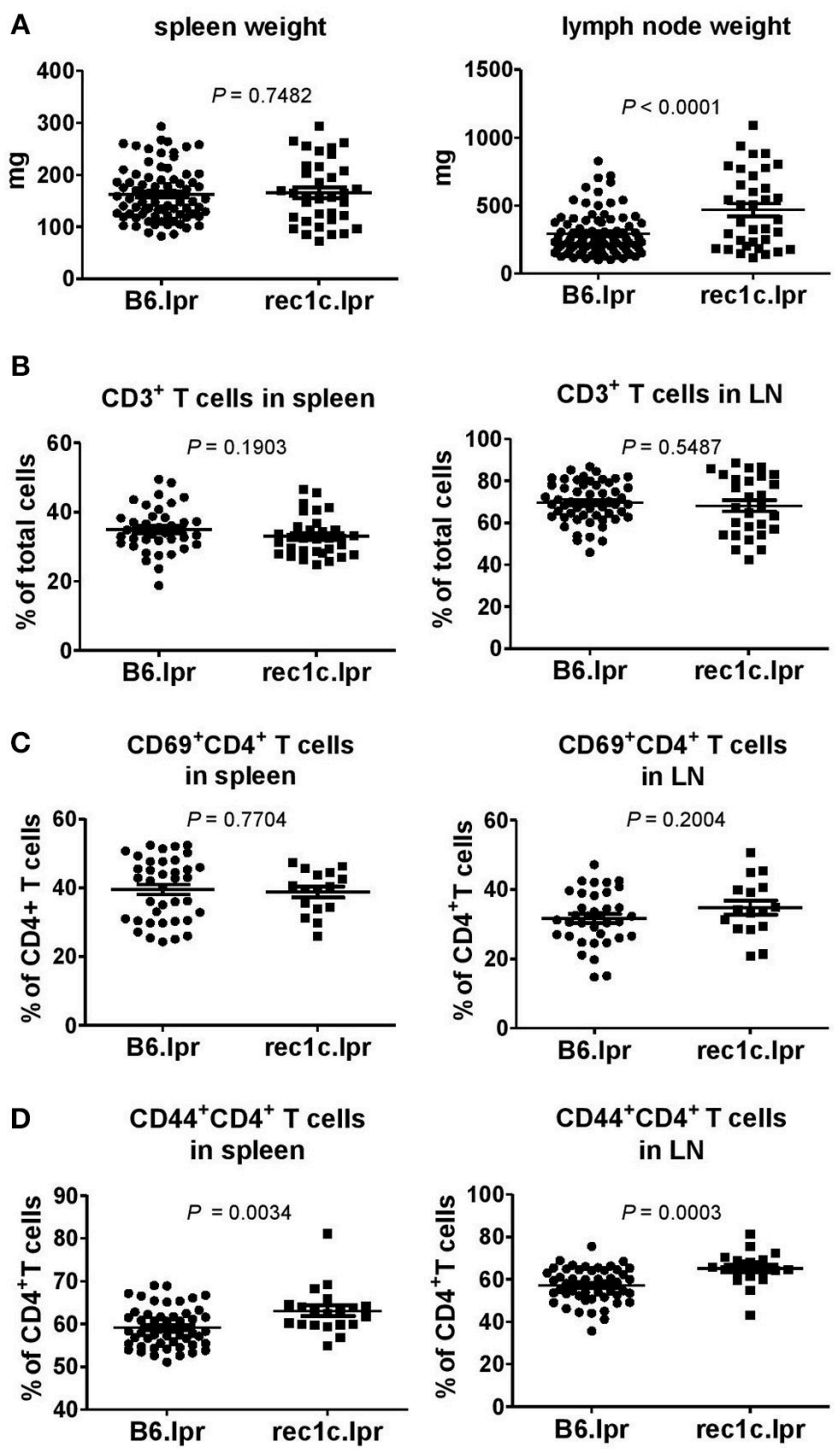
The rec1c.lpr mice show mild immune activation. Weight of spleen and lymph nodes **(A)**, and percentages of CD3^+^ T cells **(B)** as well as CD69^+^CD4^+^ **(C)** and CD44^+^CD4^+^ **(D)** T cell subsets in spleen and lymph nodes in B6.lpr and rec1c.lpr mice at age of 4–5 months. Statistical analysis was performed using two-tailed Mann-Whitney tests.

We analyzed the autoimmune pathology of the rec1c.lpr strain by comparing with control B6.lpr mice at the age of 4–6 months. The spleen sizes of rec1c.lpr mice were similar as that of B6.lpr mice, but the rec1c.lpr mice presented larger pooled lymph nodes (469 ± 46 mg), about 2 times larger than that of B6.lpr mice (292 ± 16 mg) (Figure 2A). The rec1c.lpr mice showed the same percentage of CD3^+^ T cells in spleen and lymph nodes (Figure 2B) and similar frequencies of CD4^+^ T cell expressing the early activation marker CD69 (Figure 2C) as B6.lpr mice. A small but significant increased frequency of CD44^+^CD4^+^ effector T cells was however observed in rec1c.lpr mice (Figure 2D).

**Figure 3:**
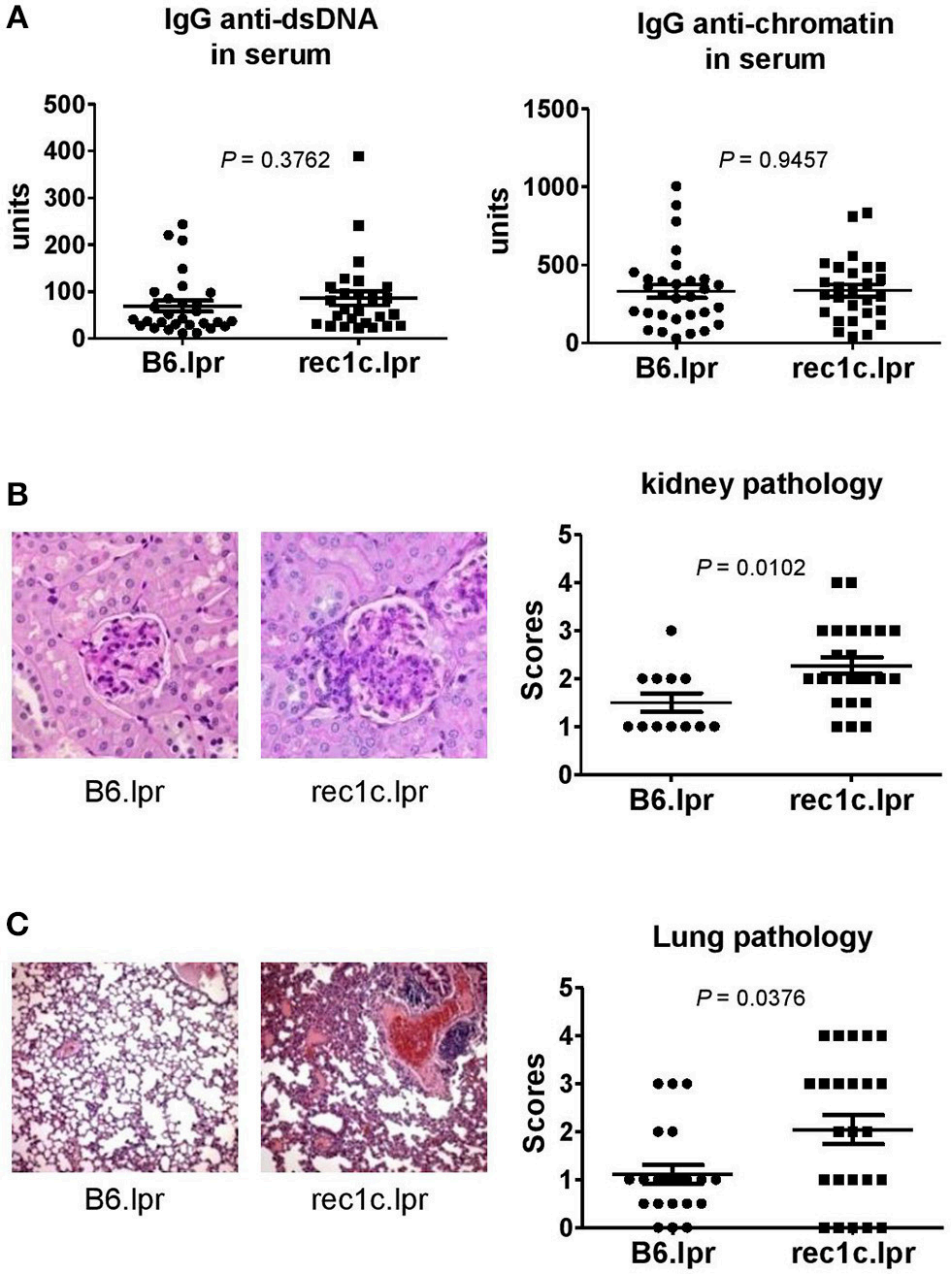
The re1c.lpr mice develop glomerulonephritis and lung inflammation. **(A)** Serum levels of IgG anti-dsDNA and anti-chromatin autoantibodies. **(B)** Representative PAS-stained kidney section (× 400 magnification) and renal histopathology scores. **(C)** Representative H&E-stained lung section (× 100 magnification) and pulmonary histopathology scores. All samples were harvested from B6.lpr and rec1c.lpr mice at age of 4–5 months. Data analysis was performed using two-tailed Mann-Whitney tests.

The rec1c.lpr mice produced the same amount of serum anti-dsDNA and anti-chromatin IgG as the B6.lpr mice (Figure 3A). As the rec1c.lpr mice displayed a milder lymphadenopathy than rec1a.lpr or rec1d.lpr mice, the pathology of their kidneys and lungs was not examined in our previous report (7). Unexpectedly, we found that rec1c.lpr mice developed significantly more severe renal and lung inflammation than age-matched B6.lpr mice (Figures 3B,C). B6.lpr mice showed a mild mesangial expansion, but the rec1c.lpr mice displayed a markedly proliferative kidney pathology with glomerular cell proliferation and inflammatory cell infiltrates in addition to mesangial expansion (Figure 3B). Most of B6.lpr mice exhibited normal blood vessels in their lungs, thin inter-alveolar septum, or a low degree of inflammatory infiltrates. In contrast, the rec1c.lpr lungs showed obvious histopathological alterations, including the presence of numerous congested blood vessels, large peribronchiolar and perivascular inflammatory cell infiltrates (Figure 3C). We also examined liver tissues and skin appearance. Most of B6.lpr or rec1.lpr mice displayed normal liver histology, although a few of them had perivascular inflammatory cell infiltrations without a difference between strains. Neither rec1c.lpr nor B6.lpr mice develop skin disease. These results indicated that the *rec1c* sublocus contains some potential disease-causing allele(s), which promotes inflammation of end organs.

### A *sNASP* Variant Allele Was Identified in the *rec1c* Interval

**Figure 4:**
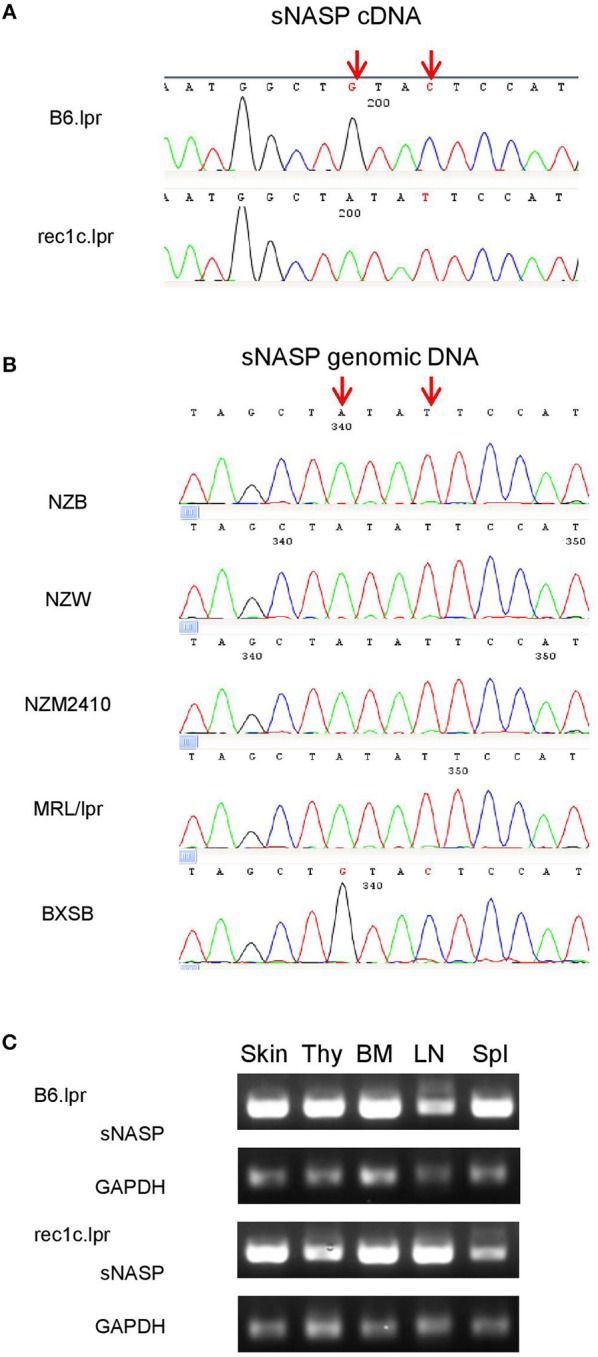
A *sNASP* variant is identified in the *rec1c* interval. DNA sequencing was performed using the Sanger method. The mutated bases of the *sNASP* allele (red arrows) are present in the rec1c.lpr cDNA **(A)** and in the exon 10 of the NZB, NZW, NZM2410, and MRL/*lpr sNASP* gene **(B)**. **(C)** *sNASP* mRNA expression in skin (Ski), thymus (Thy), bone marrow (BM), lymph node (LN), and spleen (Spl) was detected by RT-PCR. *Gapdh* was used as a control.

To uncover potentially pathogenic genetic variants in the *rec1c* interval, we sequenced all exons of its 44 protein-coding genes using whole exome sequencing (WES). As a result, we identified a variant of somatic nuclear autoantigenic sperm protein gene (*sNASP*) with two mutations in exon 10: Chr4:g116,276,661 G>A and Chr4:g116,276,664 C>T (NCBI m37 assembly), which correspond to 841G>A and 844C>T, respectively, in the *sNASP* cDNA sequence (Figure 4A). Consequently, the *rec1c* allele of the sNASP protein has a substitution of two consecutive amino acid residues, V281I and L282F, in its putative histone-binding motif (19). We therefore anticipate that the *rec1c* sNASP protein may have an altered binding to histones. Sequencing of *sNASP* exon 10 in the NZB, NZW, NZM2410, MRL/lpr, and BXSB lupus-prone mice showed the *rec1c* mutations in the NZB and NZM2410 genomes, as expected, also in the NZW and MRL/lpr strains, but not in the BXSB strain (Figure 4B). B6.lpr and rec1c.lpr mice produced comparable amount of *sNASP* mRNA in their skin, thymus, bone marrow, and spleen (Figure 4C). Overall, these results identify the *sNSAP* allele as a candidate gene for the *rec1c* interval through its possibly altered binding to histones, and show that this allele is shared among several lupus-prone mouse genomes.

### The *rec1c sNASP* Protein Is Dysfunctional in Binding Histones

**Figure 5:**
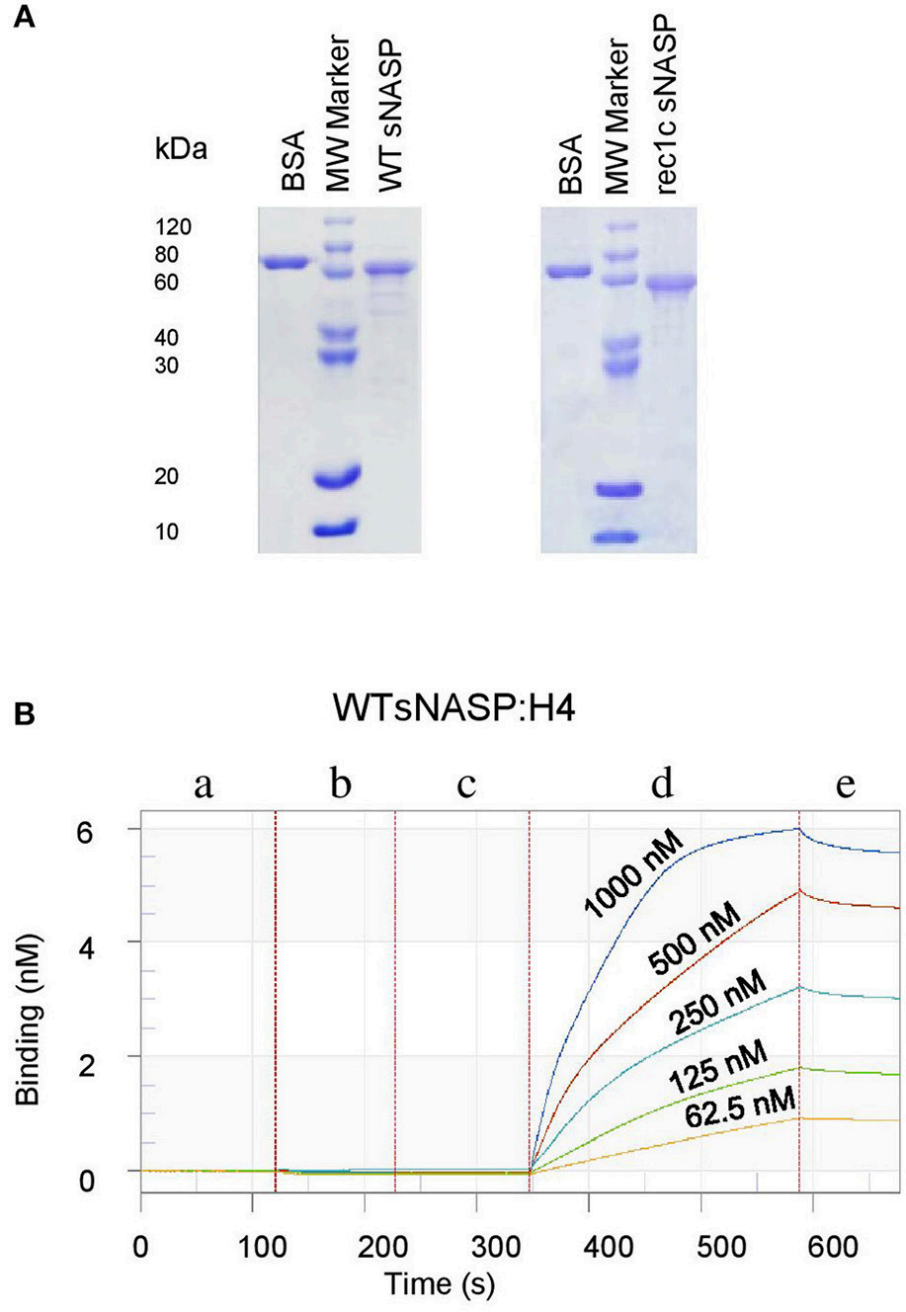
Expression, purification, and detection of histone-binding affinity of the mouse sNASP protein. Proteins were expressed in *E. coli* and purified using ion-exchange chromatography and size exclusion chromatography. **(A)** The purity of recombinant WT sNASP and *rec1*c sNASP proteins was more than 90% as confirmed by SDS-PAGE gel. Biolayer interferometer was used to detect the affinity of WT sNASP and *rec1c* sNASP proteins binding mouse histones H1a, H3.1, H4, and H3.1/H4 tetramer. **(B)** A graph of a representative assay shows the interaction of WT sNASP binding histone 4 at the indicated concentrations from 62.5 to 1,000 nM. Each experiment is represented by an initial baseline (a), a sNASP immobilization curve (b), another baseline (c), an association curve (d), and a disassociation curve (e).

We next investigated the histone-binding function of the *rec1c* sNASP protein function. WT sNASP and *rec1c* sNASP proteins were expressed in *E. coli* and purified by ion-exchange chromatography and size exclusion chromatography with more than 90% purity (Figure 5A). Quantitative binding studies of the sNASP protein interacting with mouse histones H1a, H3.1, H4, and the H3.1/H4 tetramer were measured using biolayer interferometry (BLI). A representative BLI assay graph in Figure 5B shows the interaction of WT sNASP binding H4 histone as expressed by layer thickness (nm) over time (second). Table 1 lists the binding constants of the WT sNASP and *rec1c* sNASP proteins interacting with histones H1a, H3.1, H4, and H3.1/H4 tetramer. Both WT and *rec1c* sNASP proteins showed a stronger binding affinity for H3.1 than for H1a histone, with almost a 40-fold difference. However, the WT and rec1c sNASP proteins did not show any different affinity in binding these two histones. sNASP also showed a strong binding affinity for H4 histone or H3.1/H4 tetramer in comparison with its binding to H1a histone (Table 1). The *rec1c* sNASP showed significantly lower *K*_*d*_ values for binding to H4 histone or H3.1/H4 tetramer than WT sNASP. This indicated that the *rec1c* sNASP protein has a significantly stronger affinity for binding H4 histone or H3.1/H4 tetramer than WT sNASP protein. These data demonstrate that the substitution of two consecutive amino acid residues in the *rec1* sNASP protein leads to an increased affinity of binding mouse H4 histone or H3.1/H4 tetramer.

**Table 1:** Binding kinetics and affinities for the interactions of mouse WT sNASP and *rec1c* variant sNASP proteins with mouse histones H1a, H3.1, H4, and H3.1/H4 tetramer.

	***K*_on_ (1/Ms)**		***K*_off_ (1s)**		***K*_d_ (nM)**	
	**Mean**	**SEM**	**Mean**	**SEM**	**Mean**	**SEM**	***P*-value**
**H1a BINDING**							
WT sNASP	1.006 × 10^4^	0.0281 × 10^4^	3.89 × 10^−4^	0.337 × 10^−4^	387	11.3	*p* > 0.05
sNASP variant	0.959 × 10^4^	0.0281 × 10^4^	3.95 × 10^−4^	0.353 × 10^−4^	412	12.6	
**H3.1 BINDING**							
WT sNASP	0.869 × 10^4^	0.0088 × 10^4^	1.181 × 10^−4^	0.105 × 10^−4^	13.6	1.21	*p* > 0.05
sNASP variant	0.895 × 10^4^	0.0094 × 10^4^	0.755 × 10^−4^	0.144 × 10^−4^	8.45	1.61	
**H4 BINDING**							
WT sNASP	0.199 × 10^4^	0.0010 × 10^4^	0.81 × 10^−4^	0.054 × 10^−4^	40.7	3.6	*p* < 0.01
sNASP variant	1.587 × 10^4^	0.0147 × 10^4^	3.68 × 10^−4^	0.123 × 10^−4^	23.2	0.81	
**H3.1/H4 TETRAMER BINDING**							
WT sNASP	2.44 × 10^4^	0.076 × 10^4^	9.78 × 10^−4^	0.700 × 10^−4^	40.2	3.14	*p* < 0.01
sNASP variant	3.72 × 10^4^	0.117 × 10^4^	5.39 × 10^−4^	0.549 × 10^−4^	14.5	1.54	

*Quantitative binding studies of the interactions of mouse WT sNASP and rec1c variant sNASP proteins with mouse histones H1a, H3.1, H4, and H3.1/H4 tetramer were measured using biolayer interferometer. The binding kinetic parameters were determined from four separate experiments (n = 4). Values in the table indicate mean and SEM (standard error of mean). Calculated K_d_ = k_off_ /k_on_*.

### The *rec1c sNASP* Allele Promotes Autoimmunity and Exacerbates End Organ Inflammation in a Transgenic ΔsNASP.lpr Model

**Figure 6:**
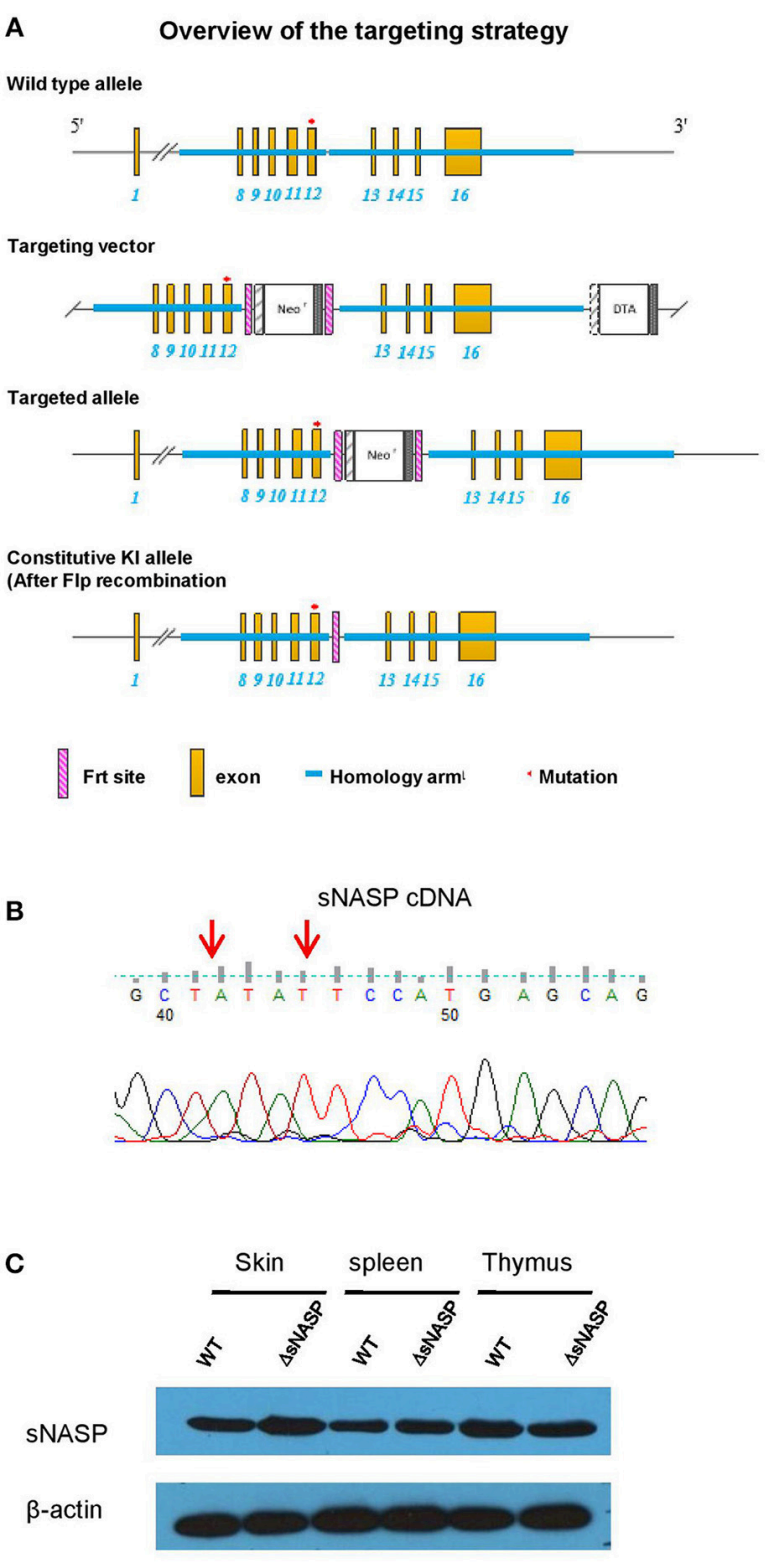
Generation of a transgenic mouse model B6.ΔsNASP expressing the *rec1c sNASP* allele. **(A)** Targeting strategy used to construct a transgenic mouse model B6.ΔsNASP in which the mutated bases of the *rec1c sNASP* allele were introduced into the B6 genome. **(B)** The *sNASP* cDNA sequence of B6.ΔsNASP mouse was verified to have the mutated bases (red arrows) of *rec1c sNASP* allele. **(C)** The sNASP protein expression was determined by Western blotting in tissues and compared between WT B6 and transgenic B6.ΔsNASP mice, and mouse β-actin was used as control.

To test the hypothesis that the *rec1c* sNASP protein, which displays an increased affinity for H4 histone and H3.1/H4 tetramer, is involved in autoimmune diseases, the most reliable approach is to construct a transgenic model with the mutated bases of the *rec1c sNASP* allele on the B6 background. Following the targeting strategy shown in Figure 6A, we used DNA homologous recombination to substitute the guanine at 4:116276661 and cytosine at 4:116276664 in the B6 genome with the corresponding adenine and thymine present in the *rec1c sNASP* allele. This transgenic model was called B6.ΔsNASP. DNA sequencing confirmed that B6.ΔsNASP mouse has the mutated bases of the *rec1c* allele in *sNASP* cDNA sequence (Figure 6B), which indicates that the *rec1c sNASP* allele was correctly introduced into B6 genome. Western blotting revealed that both B6 and B6.ΔsNASP strains express similar amounts of sNASP protein in the skin, spleen, and thymus (Figure 6C), indicating that the sNASP protein expression was not affected in the B6.ΔsNASP model. Moreover, similar to B6.rec1c mouse, the B6.ΔsNASP mouse displayed a normal growth and procreation, and did not develop any detectable autoimmune phenotypes (data not shown). Adopting the same strategy as we used with B6.rec1c.lpr, we introduced the *lpr* mutation into the B6.ΔsNASP model to generate B6.ΔsNASP.lpr (ΔsNASP.lpr) mice.

**Figure 7:**
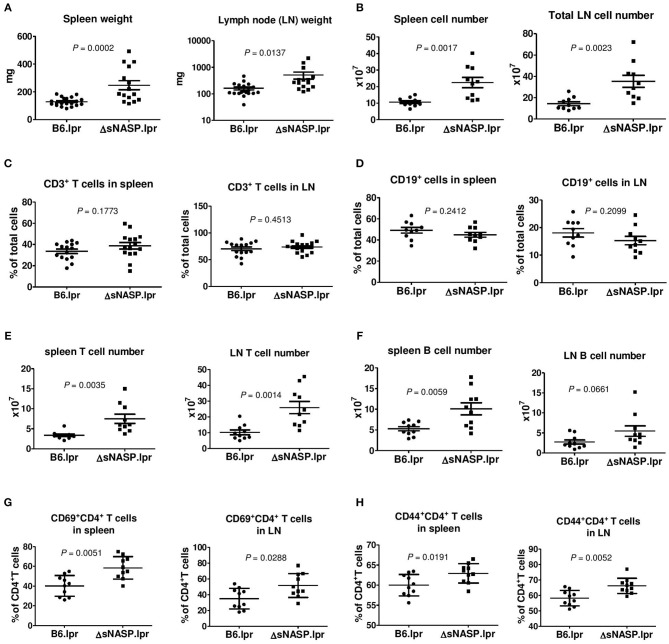
The ΔsNASP.lpr mice present activated immune phenotypes. Weight of spleen and lymph nodes **(A)** of B6.lpr and B6.ΔsNASP.lpr mice at age of 4–5 months. Total cell number of spleen and lymph nodes **(B)**. Percentages of CD3^+^ T cells **(C)** and CD19^+^ B cells **(D)**, absolute T cell number **(E)** and absolute B cell number **(F)** as well as CD69^+^CD4^+^ T cells **(G)** and CD44^+^CD4^+^ T cells **(H)** in spleen and lymph node of ΔsNASP.lpr and B6.lpr mice. Statistical analysis was performed using two-tailed Mann-Whitney tests.

We comprehensively evaluated the autoimmune phenotypes and organ pathology of the ΔsNASP.lpr as compared to B6.lpr mice at the age of 4–6 months. The ΔsNASP.lpr mice developed an enhanced lymphadenopathy with an average weight of the spleen or lymph nodes about twice and triple that of B6.lpr mice, respectively (Figure 7A). Total cell numbers in spleen and lymph node of ΔsNASP.lpr mice significantly increased in comparison with B6.lpr mice (Figure 7B). The percentages of CD3^+^ T cells (Figure 7C) and CD19^+^ B cells (Figure 7D) in spleen and lymph nodes were comparable between B6.lpr and ΔsNASP.lpr mice. However, ΔsNASP.lpr mice have more absolute numbers of splenic and LN T cells (Figure 7E) as well as splenic B cells (Figure 7F) than B6.lpr mice. In addition, ΔsNASP.lpr mice showed higher percentages of activated CD69^+^CD4^+^ T cells (Figure 7G) and effector CD44^+^CD4^+^ T cells (Figure 7H) than B6.lpr mice in spleen and lymph nodes.

**Figure 8:**
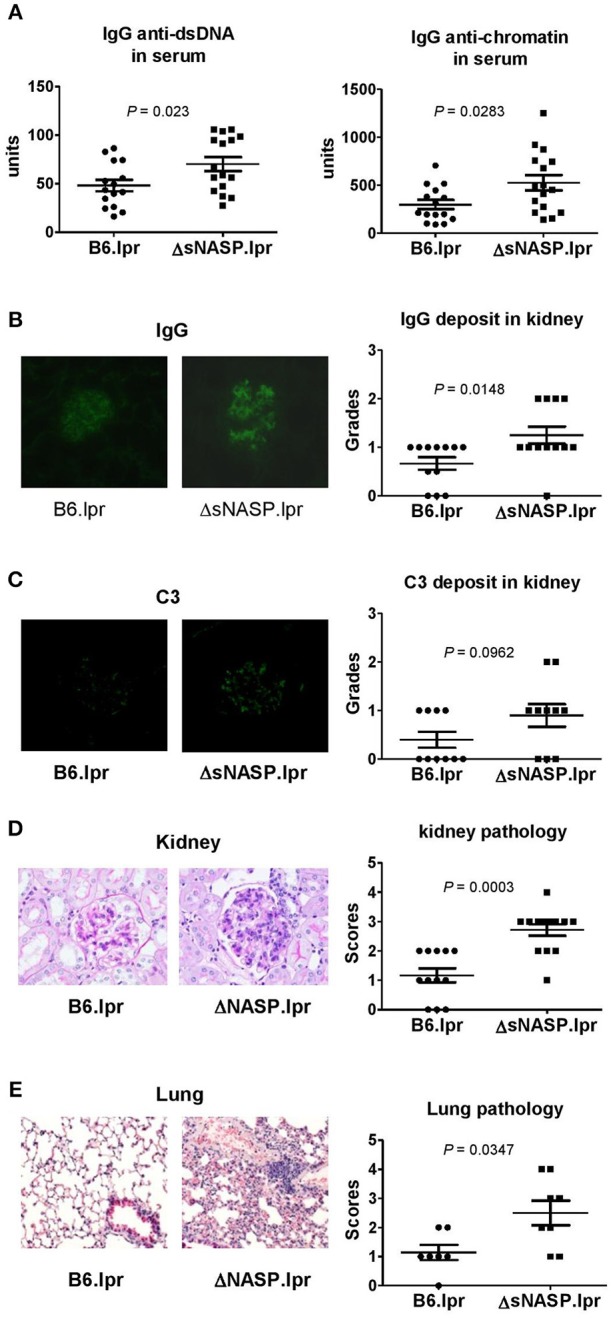
The ΔsNASP.lpr mice exhibit severe inflammatory lesions in the kidneys and lungs. Serum levels of IgG anti-dsDNA and anti-chromatin autoantibodies **(A)**. Representative images of mouse IgG **(B)** and C3 **(C)** deposit in glomeruli from B6.lpr and ΔsNASP.lpr mice (× 400 magnification) and their respective fluorescence intensity grades. Representative PAS-stained kidney section (× 400 magnification) and renal histopathology scores **(D)**. Representative H&E-stained lung section (× 100 magnification) and pulmonary histopathology scores **(E)**. All samples were harvested from B6.lpr and B6.ΔsNASP mice at age of 4–6 months. Data analysis was performed using two-tailed Mann-Whitney tests.

The ΔsNASP.lpr mice produced modestly elevated levels of serum anti-chromatin and anti-dsDNA IgG as compared with B6.lpr mice (Figure 8A). The immune complexes in kidney were detected using the indirect immunofluorescence technique. Although a small amount of mouse IgG was present in glomeruli, ΔsNASP.lpr mice showed significantly more IgG deposits in glomeruli than B6.lpr mice (Figure 8B). The ΔsNASP.lpr mice showed trace C3 deposit in glomeruli (Figure 8C). B6.lpr mice seemed to have less C3 accumulation in glumeruli than ΔsNASP.lpr mice. However, there were no statistical difference for C3 deposit in glomeruli between ΔsNASP.lpr and B6.lpr mice (Figure 8C). Pathological examination showed that the ΔsNASP.lpr mice, in addition to mild mesangial expansion, develop an enhanced proliferative renal pathology with an increased glomerular cell number and an infiltration of inflammatory cells in comparison with B6.lpr mice (Figure 8D). The renal pathology scores of the ΔsNASP.lpr mice were significantly higher than that of B6.lpr mice. However, both sNASP.lpr and B6.lpr mice at age of 4–6 months have trace proteinuria, without a significant difference between these two strains. Moreover, the ΔsNASP.lpr mice also showed significantly a more severe lung inflammation than B6.lpr mice (Figure 8E). The lung pathological characteristics of ΔsNASP.lpr mice were similar to what we observed in the rec1c.lpr mice. On the other hand, the ΔsNASP.lpr mice did not develop liver inflammation and dermatitis. In summary, the ΔsNASP.lpr mice not only reproduced all autoimmune phenotypes and organ pathology alterations of rec1c.lpr mice, but also developed additional autoimmune phenotypes, including increased sizes of spleen and lymph nodes, lymphocyte increase, expansion of activated or effector CD4^+^ T cells, IgG autoantibody elevation, and more IgG deposit in glomeruli. The characterization of the ΔsNASP.lpr model demonstrates that the *sNASP* allele is responsible for pathogenic contribution of the *rec1c* sublocus to mouse lupus.

## Discussion

The rec1c.lpr mice exhibited a normal spleen size and a modest lymphadenopathy in comparison with control B6.lpr mice, but they developed more significant kidney and lung inflammation, two end-organ manifestations of SLE. These results suggest that the *rec1c* sublocus seemingly is not involved in systemic autoimmunity, but is rather aggravating its consequences. This is a contrast to the adjacent rec1d1 sublocus since the rec1d1.lpr mice showed 3-fold expanded spleen or even 10-fold enlarged lymph node relative to B6.lpr mice, and this expansion was largely accounted for T cells, suggesting that *red1d1* contributes to lupus by targeting T cells (15). We have proposed a model for the genes involved in lupus pathogenesis with a first group of genes breaking tolerance, such as *Ly108* in *Sle1b* (23), a second group amplifying/ polarizing autoimmune activation, such as *Pbx1* in *Sle1a* (24), and a third group of genes modulating disease severity in target organs, such as the kallycrein gene family in *Sle3* (25) in the NZM2410 lupus model (26). We propose that the *Skint6* allele in *rec1d1* belong to group 2 while *sNASP* variant in *rec1c* belongs to the third group. The detailed analysis of the *Sle2c* locus revealed a complex architecture with a total of four genes so far associated with lupus susceptibility: *Cdkn2c*, which regulates B1a cell expansion, the original selecting phenotype for *Sle2c* (8), a protective allele of *Csf3r* (11), a variant of *Skint6* associated with T cell activation (15) and now a variant of *sNASP* that amplifies autoimmunity and aggravate tissue pathology. The presence of these two latter variants may explain why *Sle2*, which is not associated by itself to any end-organ pathology (27), was mapped in association with glomerulonephritis when it interacts with other NZM2410 loci (4) or with *lpr* (7).

Exon sequencing of the *rec1c* interval identified the substitution of two consecutive amino acid residues in the *NASP* gene. *NASP* contains two isoforms, a longer testis-specific *tNASP* and a shorter somatic *sNASP*. However, both isoforms often occur in transformed cell lines (28). It is well known that the sNASP functions as a histone chaperone to perform their vital role in genome maintenance by interacting with soluble histones, driving the accurate assembly and disassembly of nucleosomes (9). The substitution of two consecutive amino acid residues in the *rec1c* sNASP variant protein occurs in the histone-binding domain. We demonstrated that this variant has an increased binding affinity for histone H4 and the H3.1/H4 tetramer, suggesting that the amino acid substitutions alter its three-dimensional structure and dysfunction.

To test the functional significance of the *rec1c sNASP* variant, we introduced the corresponding two mutations into the B6 genome to generate a transgenic B6.ΔsNASP mouse and its derived ΔsNASP.lpr strain. The B6.ΔsNASP mice did not develop any detectable autoimmunity. However, the ΔsNASP.lpr mice produced more IgG autoantibodies, had bigger spleen and lymph nodes along with lymphocyte elevation, displayed mild increase of activated and effector CD4^+^ T cells in peripheral lymph organs, and more IgG deposit in glomeruli in comparison to B6.lpr mice. These phenotypes of ΔsNASP.lpr mice are a sign of autoimmunity. The ΔsNASP.lpr mice developed more severe kidney and lung inflammation than the control B6.lpr mice. Therefore, the ΔsNASP.lpr mice reproduced most of the autoimmune-pathological phenotypes of the rec1c.lpr mice. These findings establish that the *sNASP* mutant allele is responsible for the contribution of the *rec1c* interval to lupus pathogenesis. On the other hand, as the ΔsNASP.lpr mice did not develop significant proteinuria, their exacerbated kidney inflammation was not sufficient to result in renal dysfunction. As for the reason why ΔsNASP.lpr mice presented some autoimmune phenotypes different that were not found in rec1c.lpr mice, we speculate it most likely due to unlinked NZM2410 genetic contamination carried over in the rec1c.lpr congenic genome that may interfere with the *sNASP* allele. Such contamination has been documented in other NZM2410-derived congenics [(24) and Morel unpublished].

The MRL/*lpr* strain develops a rapid onset of lupus due to the *lpr* mutation in the *Fas* gene on chromosome 19. A *lpr* modifier locus, *Lprm1*, has been mapped to chromosome 4 in a genomic location close to the *rec1c* sublocus (29). We found that the MRL/*lpr* genome shares the same *sNASP* allele with the *rec1c* NZM2410 allele, suggesting that it may be responsible for the *Lprm1* phenotypes. Our study demonstrated that the *sNASP* mutant allele with higher binding affinity for histone interacts with the *lpr* mutation to modestly enhance lymphadenopathy and autoimmunity and greatly promote tissue inflammation in the ΔsNASP.lpr model. Therefore, it is reasonable to hypothesize that the interaction of the *sNASP* mutant allele and the *lpr* mutation represents an important contribution to autoimmune pathogenesis in the MRL/lpr model.

How the *sNASP* allele in the *rec1c* sublocus promotes inflammation needs to be elucidated in the future studies. We hypothesize that the increased histone-binding affinity of the *sNASP* allele may enhance the transcription of inflammatory cytokines, either by immune cells or local cells in target organs. A recent study has reported that sNASP maintains homeostasis of the innate immune response as a negative regulator of TLR signaling by binding TRAF6 and preventing its auto-ubiquitination in unstimulated macrophages (30). Following LPS stimulation, CK2 binds and phosphorylates sNASP protein at serine 158, allowing sNASP protein to dissociate from TRAF6. Free TRAF6 is then auto-ubiquitinated and participates in TLR signaling to trigger the transcription of inflammatory cytokines (30). We speculate that the sNASP variant protein in the *rec1c* sublocus may have a decreased binding affinity for TRAF6, or be more easily phosphorylated by CK2 in innate immune cells following TLR stimulation, leading to excessive TRAF6 auto-ubiquitination and inflammatory cytokine release. The *rec1c sNASP* allele may also enhance the production of inflammatory cytokines directly by facilitating access of transcriptional site or through long-range chromatin alterations. Indeed, NASP regulates chromatin accessibility by maintaining a pool of H3K9me1 methylated histones (21), an epigenetic mark associated with active transcription sites (22). Abnormal histone modification patterns have been reported in the CD4^+^ T cells of lupus patients (31). Epigenetic factors play a pivotal role in regulating cytokine expression, and hence effector functions, in lupus T cells (32). Specifically, CREMa increases IL-17A transcription (33) and the transcription factor RFX1 regulates the expression of CD11a and CD70 (34) through histone modifications in lupus CD4^+^ T cells. The critical and complex role of epigenetic regulations in lupus T cells was demonstrated by showing that specifically demethylating either CD4^+^ or CD8^+^ T cells had beneficial effects while systemic demethylation worsened disease in MRL/lpr lupus-prone mice (35). A complex pattern of DNA methylation profiles has been revealed in twins discordant for lupus with hypo-and hyper-methylation differences, including some that were cell-specific (36). Defining the mechanisms by which the histone-binding protein NASP variant contributes to lupus pathogenesis using the mouse models that we have generated, either through epigenetic alterations, or other processes such as TRAF6 activation will benefit our understanding of lupus and the regulation of inflammation in autoimmune diseases.

## Data Availability

All datasets generated for this study are included in the manuscript and/or the supplementary files.

## Author Contributions

JJ supervised the construction, genotyping, and husbandry of the transgenic B6.ΔsNASP and B6.ΔsNASP.lpr mice, and performed some experiments on B6.ΔsNASP.lpr mice. JX performed multiple pathological examinations of kidney and lung tissues of all mouse strains. YZ performed some genotyping and experiments on B6.ΔsNASP.lpr mice. XF performed recombinant vector construction and some flow cytometry experiments on B6.ΔsNASP.lpr mice. LM participated in some experimental designs, interpreted data, and wrote the manuscript. ZX designed and supervised whole project, performed experiments on rec1c.lpr mouse, and was responsible for data analysis, interpretation, and manuscript writing.

### Conflict of Interest Statement

The authors declare that the research was conducted in the absence of any commercial or financial relationships that could be construed as a potential conflict of interest.

## Abbreviations

NASP: nuclear autoantigenic sperm protein
sNASP: somatic nuclear autoantigenic sperm protein
SLE: systemic lupus erythematosus
SNPs: single nucleotide polymorphisms
ES: embryonic stem cell
KI: knockin
IPTG: Isopropyl β-D-1-thiogalactopyranoside
APS: aminopropylsilane
GN: glomerulonephritis
BLI: biolayer interferometer.
